# Somatic sequence alterations in twenty-one genes selected by expression profile analysis of breast carcinomas

**DOI:** 10.1186/bcr1637

**Published:** 2007-01-16

**Authors:** Stephen J Chanock, Laurie Burdett, Meredith Yeager, Victor Llaca, Anita Langerød, Shafaq Presswalla, Rolf Kaaresen, Robert L Strausberg, Daniela S Gerhard, Vessela Kristensen, Charles M Perou, Anne-Lise Børresen-Dale

**Affiliations:** 1 Section of Genomic Variation, Pediatric Oncology Branch, Center for Cancer Research, National Cancer Institute, National Institutes of Health, Bethesda, Maryland 20892-4605, USA; 2 Core Genotyping Facility, National Cancer Institute, National Institutes of Health, Bethesda, Maryland 20892-4605, USA; 3 Intramural Research Support Program, SAIC-Frederick, NCI-FCRDC, Frederick, Maryland 21702, USA; 4 Department of Genetics, Institute for Cancer Research, Rikshospitalet-Radiumhospitalet Medical Center, Montebello, 0310 Oslo, Norway; 5 Department of Surgery, Ullevål University Hospital, 0407 Oslo, Norway; 6 J Craig Venter Institute, Medical Center Drive, Rockville, Maryland 20850, USA; 7 Office of Cancer Genomics, National Cancer Institute, NIH, Bethesda, Maryland 20892, USA; 8 Medical Faculty, University of Oslo, 0316 Oslo, Norway; 9 Departments of Genetics and Pathology, Laboratory Medicine, Lineberger Comprehensive Cancer Center, University of North Carolina, Chapel Hill, North Carolina 27599, USA

## Abstract

**Introduction:**

Genomic alterations have been observed in breast carcinomas that affect the capacity of cells to regulate proliferation, signaling, and metastasis. Re-sequence studies have investigated candidate genes based on prior genetic observations (changes in copy number or regions of genetic instability) or other laboratory observations and have defined critical somatic mutations in genes such as *TP53 *and *PIK3CA*.

**Methods:**

We have extended the paradigm and analyzed 21 genes primarily identified by expression profiling studies, which are useful for breast cancer subtyping and prognosis. This study conducted a bidirectional re-sequence analysis of all exons and 5', 3', and evolutionarily conserved regions (spanning more than 16 megabases) in 91 breast tumor samples.

**Results:**

Eighty-seven unique somatic alterations were identified in 16 genes. Seventy-eight were single base pair alterations, of which 23 were missense mutations; 55 were distributed across conserved intronic regions or the 5' and 3' regions. There were nine insertion/deletions. Because there is no *a priori *way to predict whether any one of the identified synonymous and noncoding somatic alterations disrupt function, analysis unique to each gene will be required to establish whether it is a tumor suppressor gene or whether there is no effect. In five genes, no somatic alterations were observed.

**Conclusion:**

The study confirms the value of re-sequence analysis in cancer gene discovery and underscores the importance of characterizing somatic alterations across genes that are related not only by function, or functional pathways, but also based upon expression patterns.

## Introduction

Somatic mutations in key genes, such as oncogenes and tumor suppressor genes, have been reported to contribute to the risk for development of many human cancers. Genomic alteration has been shown to confer altered capacity for cell proliferation, metastasis, and responsiveness to either normal cellular signals or therapeutic agents [1]. Advances in sequence technology have lead to renewed efforts in the discovery and characterization of somatic mutations in different cancers. This avenue of investigation has emerged as a promising approach to dissect the profile of genetic alterations of cancer in order to better classify cancer subtypes, identify new mechanisms of carcinogenesis, and characterize possible biomarkers for susceptibility and outcome [2,3]. In fact, it is anticipated that re-sequence analysis of complete cancer genomes will be pursued in the future, because of the confluence of two major trends, the availability of complete human genome sequences, and advances in sequencing technology and analysis. Although very promising, this approach is yet to be fully developed but has been fueled in part by the characterization of somatic sequence alterations in candidate gene studies of specific cancers, such as breast and colon cancer.

Because it is still a formidable task to sequence entire genomes, investigators have analyzed individual candidate genes chosen based on results of previous studies in cell lines, animal models, or other primary human tumors. Initial re-sequence studies examined individual candidate genes based on prior genetic observations (changes in copy number or regions of genetic instability) or those identified in animal or *in vitro *laboratory studies and have defined critical somatic mutations in genes such as *TP53 *and *PIK3CA *[4-11]. To date, most studies have concentrated on coding regions and the adjacent intronic region, in search of mutations that alter the coding sequence or RNA splicing. Selected studies have extended the choice of candidate genes to include a complete gene family, such as the protein kinase family or tyrosine phosphatome [2,3,12-14]. Concentrated investigation in the protein kinase genes has been conducted because of prior evidence that selected genes, such as *PIK3CA*, are frequently mutated in breast cancer [7-11]. Stephens and coworkers [15] reported on the re-sequence analysis of the coding region of 518 protein kinase genes in breast, lung, and testicular cancer. Recently, Sjoblom and colleagues [16] surveyed somatic alterations in 13,023 genes in 11 breast and 11 colon cancer cell lines or xenografts.

The success of the candidate gene approach has provided the impetus for this study to re-sequence 21 genes chosen mainly based upon expression profiling studies. These genes vary in expression across breast carcinoma samples and have been shown to be useful for breast cancer subtyping and prognosis [17-19]. Overall, copy number of the genes was not changed in the breast tumors, as assessed by array-based comparative genomic hybridization analyses [20]. Herein, we report the re-sequence analysis in 91 primary breast tumors of coding and noncoding regions of genes drawn from a novel paradigm, which was to select genes that have an altered expression pattern across breast carcinomas.

## Materials and methods

### DNA sampling and sequence analysis

Genomic DNA from 91 tumor samples were included in this study, of which 82 were from Norwegian breast cancer cases and nine were from a new breast cancer study in North Carolina. The Norwegian breast cancer samples were selected from a series of 215 previously published primary breast cancer samples, of which 63 of the 82 included in this study have been analyzed using cDNA microarrays (Langerod and coworkers, unpublished data). Patient tissue samples were sequentially collected at Ullevål University Hospital from 1990 to 1994 under an institutional review board approved protocol.

Primary breast carcinoma tissue was snap frozen and stored at -80°C. Frozen sections stained with hematoxylin/eosin were reviewed to confirm tumor content. More than 80% of the samples analyzed had more than 40% tumor cell content. Blood samples were collected in EDTA tubes and frozen at -40°C before DNA was isolated. DNA was extracted from both peripheral blood cells and tumor tissue using a method of chloroform/phenol extraction followed by ethanol precipitation (Nuclear Acid Extractor 340A; Applied Biosystems, Foster City, CA, USA), according to standard procedures. Matched control genomic DNA was available from peripheral blood from 36 of the Norwegian breast cancer cases.

**Figure 1:**
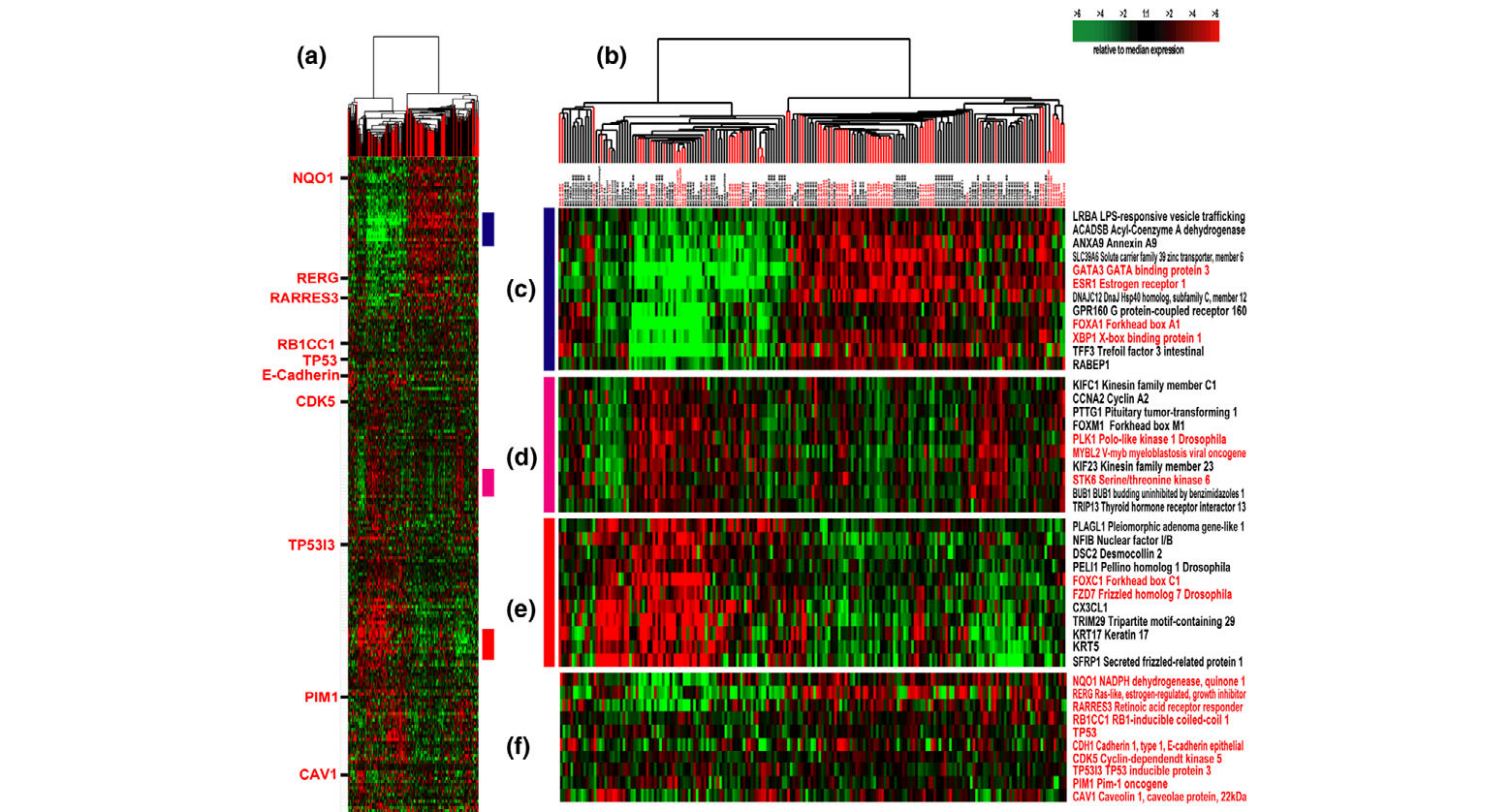
Hierarchical clustering analysis of 194 breast tumor samples analyzed using the 'SAM264' patient survival associated gene set augmented with nine additional genes included in the resequencing analysis. **(a) **Hierarchical clustering overview that shows the overall context for the 21 genes. **(b) **Close up of the sample associated dendrogram, which identifies the tumor samples that were re-sequenced in red. **(c) **Luminal/ER^+ ^epithelial gene set showing coordinated expression of *ESR1*, *GATA3*, *FOXA1*, and *XBP1*. **(d) **Proliferation gene set showing expression of *STK6*, *MYBL2*, and *PLK1*. **(e) **Basal epithelial gene set showing the expression of *FOXC1 *and *FZD7*. **(f) **The expression profiles of the additional genes that were re-sequenced but that did not fall into the previous expression patterns are shown, and their position in the larger cluster is also shown in panel a. All genes identified in red text were analyzed by re-sequencing in this study, and only *FBXW7 *and *PIN1 *were not included in this cluster analysis because their average expression levels did not meet the gene filtering criteria. ER, estrogen receptor.

Of the set of 21 genes selected for this re-sequencing analysis, 13 of them (*FZD7*, *NQO1*, *MYBL2*, *PLK1*, *STK6*, *ESR1*, *FOXA1*, *FOXC1*, *GATA3*, *RARRES3*, *RERG*, *XBP1*, and *CDK5*) were selected primarily based on their variation in gene expression patterns from previous studies of breast carcinomas [17,18] and eight were selected based on previous reports that they harbor somatic mutation in breast cancers (*CAV1*, *CDH1*, *FBXW7*, *PIM1*, *PIN1*, *TP53*, *TP53I3*, and *RB1CC1*), although they showed considerable variation in expression patterns (Figure 1 and Additional file 1).

Sequencing primers were designed for bidirectional sequence analysis using Primer3 software [21]. Each oligonucleotide was extended with a universal sequencing primer: M13 forward (TGTAAAACGACGGCCAGT) or M13 reverse (CAGGAAACAGCTATGACC). Primers and conditions are posted on the SNP500 Cancer website [22]. Standard cycle sequence analysis was performed (MJ Research PTC-200 Thermacycler) (MJ Sciences Waltham, MA, USA). Polymerase chain reaction (PCR) products were cleaned up with Exonuclease I/Shrimp Alkaline Phosphatase (USB, Cleveland, OH, USA). PCR products were sequenced using a modified ABI Prism^® ^BigDye Terminator protocol (ABI, Foster City, CA, USA). Pgem^®^-3Zf(+) (Promega Corp., Madison, WI, USA) was used for controls in all sequencing reactions. The sequencing reactions were cleaned up by either Sephadex G-50 (Sigma, St Louis, MO, USA) spin columns in a MultiScreen^®^-HV 96-well filter plate (Millipore, Billerica, MA, USA) or Performa^® ^DTR 384-well spin plate (Edge BioSystems, Gaithersburg, MD, USA). The reactions were run on either ABI 3700 or ABI 3730XL (ABI). Sequence traces were reviewed by two independent reviewers.

Bidirectional sequence analysis included 166 exons (62,000 bp) and an additional 120,000 bp of noncoding sequence in tumor samples from the 91 cases of breast cancer. In each gene, sequence analysis targeted at least 2 to 3 kb upstream of the first exon and 2 kb downstream of the 3'-untranslated region, as well as evolutionarily conserved intronic regions (defined as 75% or greater sequence similarity over a 200 bp fragment for alignment of mouse and human sequence) [23]. For each amplicon in which a sequence variant was observed in a tumor sample, sequence analysis was also performed in the SNP500 Cancer reference set of 102 individuals drawn from the four major ethnic groups of the USA and a set of 94 anonymized Norwegian women who were older than 55 years and had a history of two negative mammograms [24].

We estimated the mutation rate based on the number of somatic events observed divided by the total number of base pairs sequenced in the analysis. This calculation combines potentially functional mutations (for example, driver mutations) with passenger somatic alterations [15].

### Hierarchical clustering analysis

A total of 194 breast tumors were analyzed by clustering analysis using a modified version of the 'SAM264' gene list [18]; the 'SAM264' gene set is the set of genes that were associated with survival as identified using a Significance Analysis of Microarray analysis and contained 10 of the 21 genes sequenced. We added the 11 remaining re-sequenced genes to the SAM264 list for clustering analysis. Initially, we created a single sample set that was a combined dataset of the previous 122 samples [18,19], and 63 tumors from Langerod and coworkers (unpublished data) and nine tumors from North Carolina that included most of the samples used for the resequencing analysis presented here. This combined sample set was used to guide gene selection for resequencing analyses.

Because these three sets of samples were assayed using different microarrays, the two-color cDNA microarray datasets [18,19] (Langerod and coworkers; unpublished data) and the nine Agilent A1 microarray experiments performed at University of North Carolina, they were pre-processed similarly and systematic array biases removed using distance weighted discrimination (DWD) [25]. First, gene annotation from each dataset was translated to UniGene Cluster IDs (Build #185) using the SOURCE database [26]. The pre-processing included an initial selection for genes that exhibited a signal intensity of greater than 30 units in both the Cy3 and Cy5 channels across at least 70% of the experiments, which caused *FBXW7 *and *PIN1 *to be removed from further analyses because of very low signal intensities. Next, we log^2 ^transformed the R/G ratio and then Lowess normalized the data [27]. Missing values were imputed using the k-NN imputation algorithm (*k *= 10) described by Troyanskaya and coworkers [28]. The expression values for duplicated probes with the same Unigene cluster ID were collapsed using the median expression value. DWD was performed in a pairwise manner by first combining the dataset reported by Sørlie and coworkers [18] with that by Langerd and coworkers (unpublished data), and subsequently combining this with the University of North Carolina data. In the final step of pre-processing, each individual experiment (microarray) was normalized by setting the mean to zero and its standard deviation to one, and each gene was median centered. The DWD corrected data for the SAM264 genes plus nine additional genes was finally used in a two-way average linkage hierarchical cluster analysis using centered correlation across the 194 microarrays.

## Results

**Figure 2:**
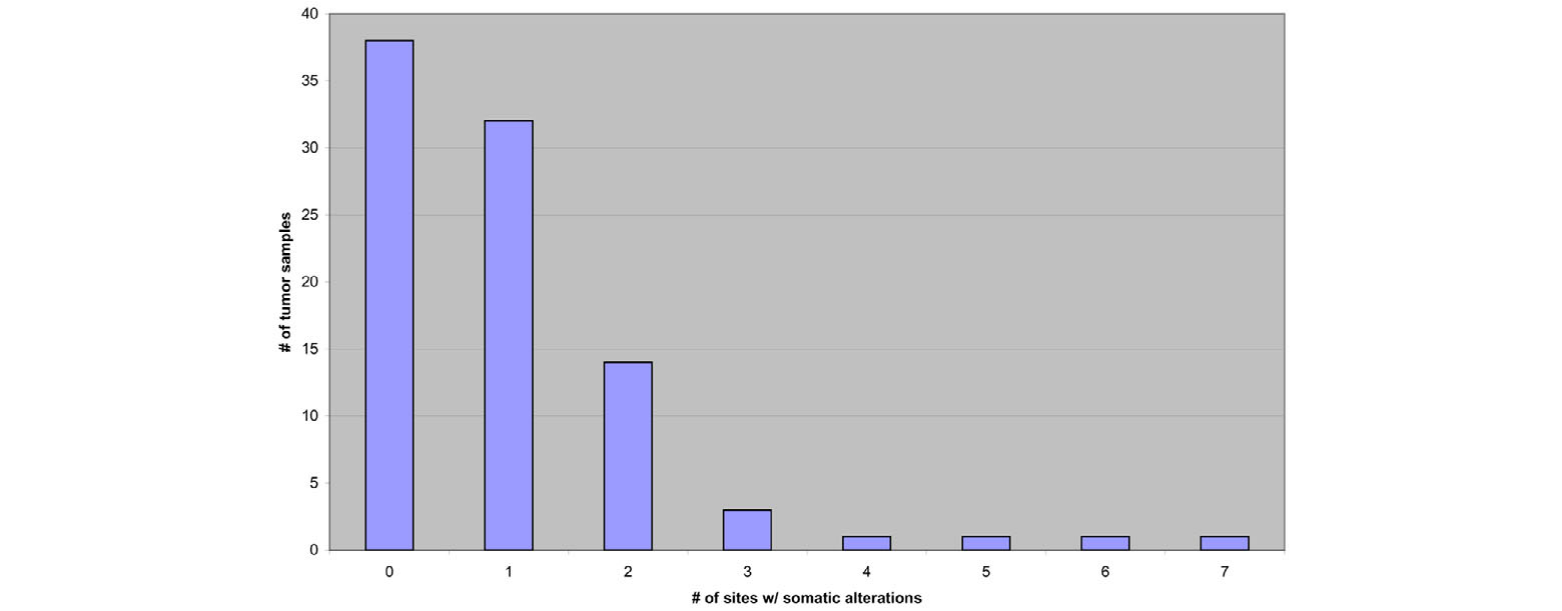
Distribution of samples with observed number of somatic alterations. Total number of somatic alterations per sample are included underneath the bars and the total number of samples in each category is represented on the vertical axis.

In total, more than 16.2 megabases were sequenced and more than 95% of the targeted amplicons were analyzed. Eighty-seven unique somatic nucleotide variants were identified in 16 genes (*TP53*, *GATA3*, *CAV1*, *CDH1*, *ESR1*, *FBXW7*, *FOXA1*, *FOXC1*, *FZD7*, *MYBL2*, *PIN1*, *RB1CC1*, *RERG*, *STK6*, *TP53I3*, and *XBP1*; see Table 1 and Additional file 2 for detailed results for each sample). In five genes (*CDK5*, *NQO1*, *PLK1*, *PIM1*, and *RARRES3*) no somatic sequence alterations were observed. The majority of sequence alterations were observed once, although there were three missense mutations that were observed more than once. The distribution of the somatic alterations per tumor sample is shown in Figure 2. Fifty-three tumors had one or more somatic alteration, and in 38 tumor samples (42%) no somatic alterations were noted. The largest number of somatic alterations observed was seven in one sample, but these were distributed over four separate genes. The overall distribution of the single base pair somatic alterations favored transitions over transversions (50 versus 28).

Of the 87 total somatic alterations observed, 78 single base pair somatic alterations were distributed across 16 of the 21 genes analyzed (Table 1). For the purposes of this analysis, a single base pair somatic alteration was defined on the basis that it was observed in a tumor specimen but not in the constitutional DNA of 102 controls from the SNP500 Cancer set, or matched blood DNA of 36 of the Norwegian breast cancer cases. Of the 78 single pair somatic alterations, we observed 34 alterations in coding regions; 23 were missense alterations and 11 were predicted to be synonymous changes (for example, no alteration of the predicted amino acid). In noncoding regions, 44 single base pair somatic alterations were observed, of which 27 were in evolutionarily conserved intronic regions, 12 in the analyzed 5' region, and five in the analyzed 3' region.

Sequence analysis of the tumor samples identified 252 single nucleotide polymorphisms (SNPs), all of which were confirmed in blood samples drawn from 94 Norwegian women with no history of breast cancer and the reference SNP500 Cancer set [22].

We observed 23 missense mutations in eight of the 21 genes studied. *TP53 *and *GATA3 *were notable because of the large number of sequence alterations observed, which included missense mutations and insertion/deletions previously reported [4-6,29]; in total, there were 14 distinct missense mutations, one pre-terminal stop codon, four insertion/deletions, and five noncoding alterations. For both of these genes, the majority of sequence variants have been shown to be functionally significant somatic mutations, and thus could be considered as 'driver' mutations for oncogenesis [15]. Eight novel missense alterations were found in six additional genes (*CDH1*, *FBWX7*, *ESR1*, *RB1CC1*, *TP53I3*, and *XBP1*). Of the eight missense alterations, two were observed in *CDH1 *and two in *ESR1*, and four overall resulted in significant amino acid shifts by Miyata criteria [30]. In *RB1CC1*, a significant amino acid shift is predicted, namely R1514C, with a high Miyata score of 3.06; this results in a positively charged residue being changed to a hydrophilic residue. The mutation M180K in *TP53I3 *has a Miyata score of 2.63 and predicts a change from a hydrophobic to a positively charged residue. In *ESR1*, a H6Y with a Miyata score of 2.27 predicts a shift of a positive charge to a hydrophilic charge. In the *FBXW7 *gene, the E117K substitution with a Miyata score of 1.14 results in a shift from negative to positive charge. There are several conservative substitutions that have low Miyata scores: in the *CDH1 *gene the M282I variant was observed twice and gave a Miyata score of 0.29, and the D777N variant has a Miyata score of 0.65; in *ESR1 *the M264I variant has a score of 0.29; and in *XBP1 *the variant R232K has a Miyata score of 0.4.

In *FZD7*, we observed two synonymous variants, L23L and L26L, which are both in close proximity to a common SNP in codon 24 that results in a conservative shift from glycine to arginine. Notably, a second SNP that also affects codon 24, namely G24S, was seen in the Norwegian population, which also results in a conservative shift with a Miyata score of 0.85. These data suggest that this could be a region of increased mutational activity, but further work on breast tumors and cell lines is needed to characterize the functional implications of the changes. The distribution of alterations did not differ from that of the SNPs across the same regions for both coding and noncoding regions.

Insertion/deletion somatic alterations were observed in four genes, and there were a total of nine. Six were insertion/deletion alternations within the coding region of the gene and one, a 4 bp insertion, occurred at the splice site junction in *CDH1*. The gene most frequently observed with insertion/deletion (four) was *CDH1*, which has previously been reported to have altered copy number (loss of heterozygosity), and can undergo somatic mutation and silencing by methylation [31-33]. Mutations in *CDH1*, particularly frameshift mutations, are seen more frequently in the lobular histologic subtype [34], and in our series two out of three with frameshift somatic alterations were observed in tumors classified as lobular.

Of the 21 genes included in the re-sequencing analysis, 10 of the 21 were contained within the 'SAM264' set of genes, which represents genes that were associated with breast cancer patient survival times [17]. In order to visualize the expression patterns of all the re-sequenced genes, we added the 10 missing genes (*CDH1*, *CAV1*, *FBXW7*, *PIM1*, *PIN1*, *TP53*, *TP53I3*, *RB1CC1*, *FZD7*, and *CDK5*) to the SAM264 gene set and performed a hierarchical clustering analysis using a dataset of 194 tumors, which was the combined data on the tumors used to select genes for re-sequencing analysis [18,19] and 72 of the tumors that were actually re-sequenced (Figure 1). After standard data quality gene filtering methods were employed, 19 out of 21 genes were present (*FBXW7 *and *PIN1 *gave very low signal intensities) in the clustering analysis and clearly exhibited significant variation in expression across the 194 breast tumors (Figure 1). A clustering analysis using the modified SAM264 list and just the 63 Norwegian samples that were included in the re-sequencing was also performed (Additional file 1), and in this analysis the differential expression of the genes over this set of tumor samples recapitulated our previous findings and showed that many of the re-sequenced genes have expression patterns that define the breast tumor subtypes [18,19].

## Discussion

We report the results of a re-sequence analysis of 21 candidate genes in 91 primary breast tumors. The candidate genes were chosen mainly based upon previous expression profiling studies and the target sequencing regions were extended beyond coding regions to include evolutionarily conserved regions and the 5' and 3' regions. This latter point is essential because it underscores the importance of examining genetic regions that could alter the expression or stability of a gene. Our results identified a spectrum of single base sequence alterations in 16 of the 21 genes selected for targeted re-sequence analysis.

Unlike the report by Stephens and coworkers [15], we did not observe clustering of sequence alterations in a single tumor. The maximum number of alterations in any of the 21 genes observed in a single tumor was seven, and we observed no somatic alteration in nearly 40% of the samples analyzed. We can exclude the likelihood that loss of heterozygosity could account for this, because the density of common SNPs observed across the 21 genes was comparable to the density observed in the SNP500 Cancer set and the International HapMap study [35]. We observed a comparable ratio for transitions to transversions to that reported by Stephens and colleagues [15].

In our analysis we observed 27 total somatic alterations mutations in eight of the 21 genes studied, and of these 23 were unique missense alterations. In contrast, there were 11 synonymous alterations. The difference in the observed number of nonsynonymous changes relative to synonymous SNPs did not deviate significantly from expected [36]. Unlike the study conducted by Stephens and coworkers [15], we did not observe enrichment of nonsynonymous changes relative to synonymous changes in our set of 21 genes chosen on the basis of expression profiles.

Wide variance in the number of somatic alterations per gene was observed, which did not always correlate with previous reports. For instance, in the *RB1CC1 *gene, which was previously reported to undergo truncating mutations in breast tumors [37], our bidirectional sequence analysis revealed eight noncoding alterations, two synonymous alterations, and a single nonsynonymous change, namely R1514C (Miyata score of 3.06), which results in a positively charged residue shift to a hydrophilic residue. In *PIN1*, a gene previously reported to be mutated in breast cancer [38], we observed only one synonymous nucleotide change. Interestingly, two alterations were observed in *FBXW7*, namely a nonsynonymous E117K with a significant amino acid shift and an intronic alteration; this is in contrast to previous reports [39,40], which found a higher rate of mutation in *FBXW7 *in breast cancer cell lines.

Our approach differed from the prior reported studies in that the re-sequence analysis also targeted regions of possible regulatory importance. In fact, our study targeted sequence in contiguous noncoding regions, which could be enriched for regulatory regions to be defined functionally. Because there is no *a priori *way to predict whether any one of the identified synonymous and noncoding somatic alterations disrupts function, analysis unique to each gene will be required to establish whether the change is functional. At this time, we interpret the majority of these changes to be nonfunctional and perhaps related to an increase in background mutation rate in cancer, specifically in breast cancer. In this regard, we confirmed the findings of Stephens and coworkers [15] that the majority of observed somatic variants are probably passenger or hitchhiking mutations and not necessarily subject to selection. Further laboratory work is needed to determine which sequence alterations bear functional consequences for the development of breast cancers.

It is notable that we observed somatic alterations in genes not observed in the study conducted by Sjoblom and coworkers [16] (for instance, *ESR1*, *GATA3*, and *CDH1*). This is not surprising because our study included a large number of estrogen receptor positive and estrogen receptor negative samples in our SNP discovery phase. Moreover, *GATA3 *and *ESR1 *mutations appear to be mutated primarily in estrogen receptor positive tumors, which were not included in the discovery cell line set used in the study conducted by Sjoblom and colleagues [29].

The prevalence of somatic mutations probably varies between different cancers and possibly by populations [2,3,14,15]. Previous surveys of the coding regions in colon and breast cancer were biased toward genes of the protein kinase family and reported a rate of approximately one somatic alteration per megabase of sequence. We estimate that the rate of somatic mutation in genes altered in expression pattern in breast cancer is slightly higher than that reported for colon cancer and breast cancer [2,3,12]. Based on this survey of coding and noncoding sequence (at a ratio of 1:3) for 21 genes, we estimate the rate of somatic alteration could be as high as 5.3 per megabase, but this assumes that all variants are indeed somatic variants. It is possible that a subset could be rare germline variants. Thus, our estimate for the somatic mutation rate in breast cancer is slightly higher than the previous reports of approximately 1.2 nonsynonymous somatic changes per megabase in colon cancer [2,3,15]. We also note that two-thirds of the sequence analyzed in the present study is noncoding. An estimate of the rate of somatic alterations did not differ between noncoding and coding regions, suggesting that the majority could be hitchhiking mutations. In an analysis of 11 breast cancer cell lines and colon tumor xenografts, Sjoblom and coworkers [16] also observed more somatic alterations, approximately 2.5 more, than in the earlier studies [2,3,15]. Together with our results, these data suggest that somatic alterations could arise more frequently than was originally reported. It is also notable that our study also targeted noncoding regions, where somatic alterations rates might differ from those in coding regions. Further studies are required to address this important point.

It is plausible that our study might also underestimate the rate of somatic alteration because we previously identified five additional mutations in the *TP53 *gene [6] (Langerod and coworkers, unpublished data) in the Norwegian tumor samples using a highly sensitive screening technique, namely temporal temperature gel electrophoresis (TTGE), prior to sequencing (these mutations are marked in Additional file 2). This prescreening allowed us to detect mutations in a heterogeneous tumor sample with as low as 1% mutated cells [41], and aberrant migrating band on the TTGE gel can be sequenced directly to define the sequence alteration. Microdissection before sequencing will not fully avert this problem because of tumor heterogeneity. To use TTGE as pre-screening is impractical because it is labor intensive to establish and not easily amenable to high throughput analyses. Because we had previously performed TTGE for *TP53 *analyses, we were able to assess the sensitivity of the different techniques; both techniques failed to identify all mutations.

## Conclusion

Systematic re-sequence analysis of a sufficiently large set of tumor samples drawn from well designed clinical and epidemiologic studies promises to identify new cancer associated genes and somatic mutations that are linked to response to cancer therapy [42-44]. The present study confirms the value of re-sequence analysis in cancer gene discovery and underscores the importance of characterizing somatic alterations across genes related not only by function, or functional pathways, but also based upon expression patterns. Advances in sequencing technology will certainly accelerate the characterization of somatic alterations in the cancer genome, but the task of defining the importance of observed somatic changes will continue to rest on the shoulders of future laboratory investigators.

## Abbreviations

bp = base pairs; DWD = distance weighted discrimination; PCR = polymerase chain reaction; SNP = single nucleotide polymorphism; TTGE = temporal temperature gel electrophoresis.

## Competing interests

The authors declare that they have no competing interests.

## Authors' contributions

SJC conceived the project, analyzed data, and wrote the paper. LB analyzed sequence tracings and managed the dataset. MY analyzed data. VL analyzed sequence tracings and managed the dataset. AL handled samples and analyzed data. SP performed experiments. RK collected clinical samples and analyzed data. RLS analyzed data and revised the paper. DSG analyzed data and revised the paper. VK collected samples, analyzed the data, and revised the paper. CMP conceived project, analyzed the data, and revised the paper. ALBD conceived the project, collected samples, analyzed the data, and revised the paper.

## Supplementary Material

Additional file 1 A pdf file showing a hierarchical clustering analysis based on the 63 breast tumor samples from Norway that were used for the re-sequencing analysis, which was clustered using the augmented 'SAM264' patient survival associated gene set. (a) Hierarchical clustering overview that shows the overall context for the 19 genes. (b) Close up of the sample associated dendrogram. (c) Basal epithelial gene set showing the expression of *FZD7*. (d) Proliferation gene set showing expression of *STK6*, *MYBL2*, and *PLK1*. (e) Luminal/ER^+ ^epithelial gene set showing coordinated expression of *ESR1*, *GATA3*, *FOXA1*, and *XBP1*. (f) The expression profiles of the additional genes that were re-sequenced but that did not fall into the previous three expression patterns are shown, and their position in the larger cluster is also shown in panel A. All genes identified by red text were analyzed by re-sequencing in this study, and only *FBXW7 *and *PIN1 *were not included in this cluster analysis because their average expression levels did not meet the gene filtering criteria. Click here for file

Additional file 2 A doc file in which observed somatic alterations are reported by individual breast cancer tissue sample (*n *= 53). Somatic alterations previously reported in *TP53 *and *GATA3 *are highlighted [4-6,29] (Langerod and coworkers, unpublished data). Click here for file

## Figures and Tables

**Table 1:** Somatic alterations by region in breast cancer re-sequence analysis

Gene	Nonsynonymous	Synonymous	5'	IVS	3'	Indel
TP53^a^	R110P, F113V, A138V (2), Y163C, Y163H, H193T, I195T, V216M, S241A, R249M, I251S, D259Y, R273C (3)			IVS1+75A>G IVS6-2A>G IVS7-1G>T		7 bp @ G286 22 bp @ L189
GATA3^a^	R366X, R366L			IVS2-281C>G	Ex5+311C>G	CA @ IVS3-3 A @ T315
*CAV1*		D143D	-2768A>C -1446T>A -596A>G		Ex3-3G>C	
*CDH1*	D777N M282I (2)	A563A		IVS3+128T>C IVS3+260A>G IVS7+47T>C IVS7+2049T>A IVS8-175G>A		CCGG @ Ex3+19 A @ Ex345 G @ Ex7+15 AAGT @ IVS13+3
*ESR1*	H6Y M264I		Ex1+139G>C		Ex8+2144T>A	
*FBXW7*	E117K			IVS1-1641A>C		
*FOXA1*			-3717G>A			
*FOXC1*			-2713G>T -1770C>T		+936G>A +940G>A	
*FZD7*		L23L L26L G409G	-1387G>C			
*MYBL2*				IVS7+15A>G IVS8-14C>G IVS12+28G>C		
*PIN1*		S38S		IVS2+3447G>A IVS2+3419G>A IVS2+3370T>C		
*RB1CC1*	R1514C	S1424S L1511L	-31467C>A -30224C>G	IVS1+5248T>G IVS1+6068A>G IVS11+97T>C IVS11-36C>T IVS15+1535C>G IVS21-16G>A		
*RERG*			Ex1+63G>T	IVS2+27438T>G IVS2-30415C>T		CTTdel @ IVS2-7163
*STK6*^b^		A172A E175E		IVS4-35A>G IVS9-33A>G		
*TP53I3*	M180K	P102P				
*XBP1*	R232K		-2339G>C	IVS4-11G>A		

Eighty-seven unique somatic alterations were identified by sequence analysis in 16 of 21 genes analyzed (*TP53*, *GATA3*, *CAV1*, *CDH1*, *ESR1*, *FBXW7*, *FOXA1*, *FOXC1*, *FZD7*, *MYBL2*, *PIN1*, *RB1CC1*, *RERG*, *STK6*, *TP53I3*, and *XBP1*). No somatic alterations were detected in five genes (*CDK5*, *NQO1*, *PIM1*, *PLK1*, and *RARRES3*). All alterations were observed singly in bidirectional sequence analysis; numbers in parentheses indicates the number of unique tumor samples with somatic alterations. Intronic analysis restricted to regions including 100 bp on either side of exonic junctions and evolutionarily conserved regions between mouse and human (>75% similarity over 200 bp). ^a^Most of the nonsynonymous mutations and deletions in *TP53 *and *GATA3 *were previously reported [4,19]. ^b^In the analysis of *STK6 *(also known as *STK15*), additional alterations were observed at six sites within the target 5' region but lie in the adjacent gene *CSTF1 *(-7698C>T, -7648A>G, -7105C>T, -5992A>G, -4868A>G, and -4221G>A). None of the variants result in nonsynonymous alterations in the coding region of *CSTF*.
