# Supplementary figures and images for: Somatic sequence alterations in twenty-one genes selected by expression profile analysis of breast carcinomas
Source: Breast Cancer Res. 2007 Jan 16;9(1):R5. doi: 10.1186/bcr1637 (PMC1851401; doi:10.1186/bcr1637)

Supplemental Figure 1

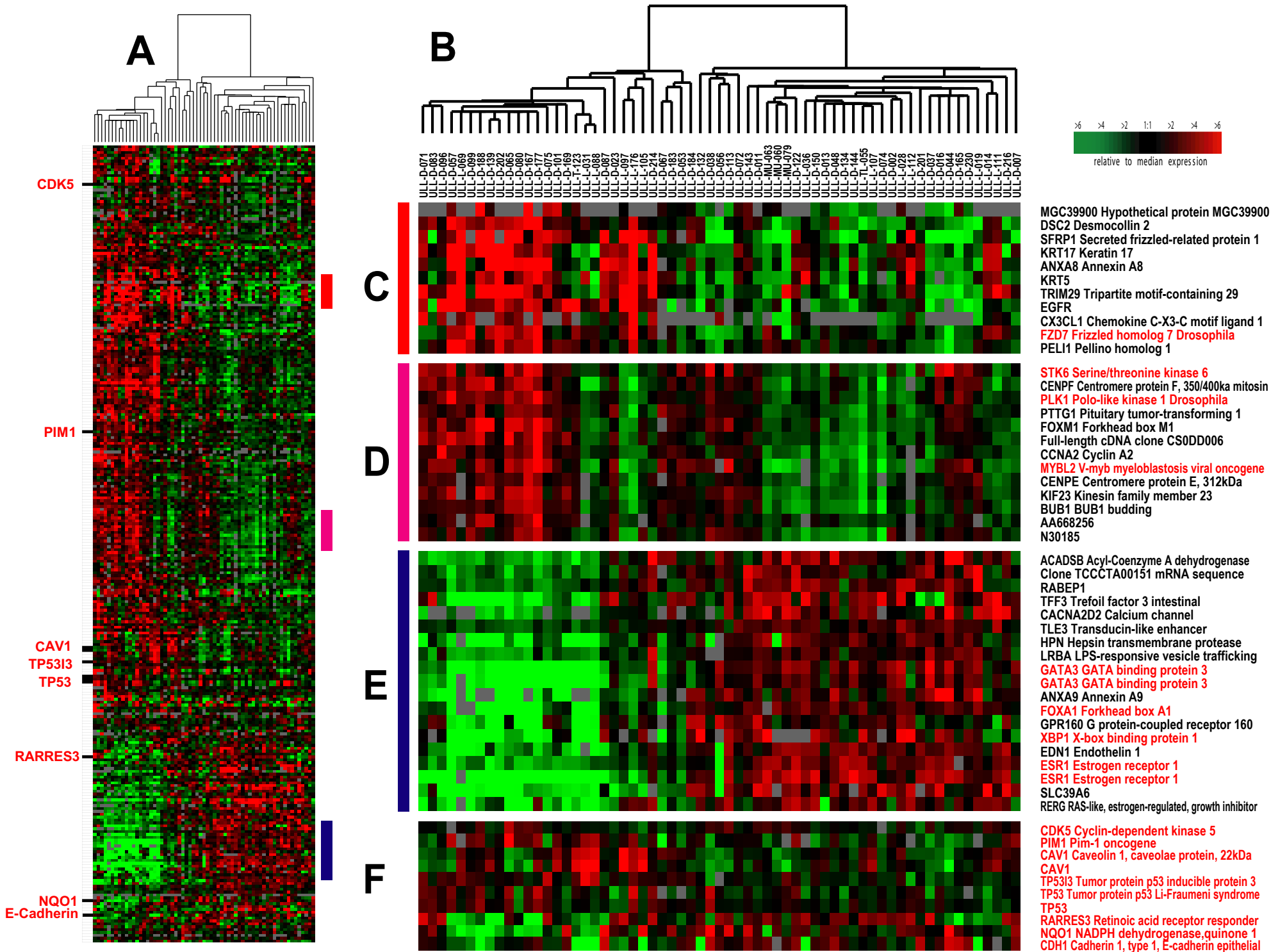

Supplement: Additional file 1 — A pdf file showing a hierarchical clustering analysis based on the 63 breast tumor samples from Norway that were used for the re-sequencing analysis, which was clustered using the augmented 'SAM264' patient survival associated gene set. (a) Hierarchical clustering overview that shows the overall context for the 19 genes. (b) Close up of the sample associated dendrogram. (c) Basal epithelial gene set showing the expression of FZD7. (d) Proliferation gene set showing expression of STK6, MYBL2, and PLK1. (e) Luminal/ER+ epithelial gene set showing coordinated expression of ESR1, GATA3, FOXA1, and XBP1. (f) The expression profiles of the additional genes that were re-sequenced but that did not fall into the previous three expression patterns are shown, and their position in the larger cluster is also shown in panel A. All genes identified by red text were analyzed by re-sequencing in this study, and only FBXW7 and PIN1 were not included in this cluster analysis because their average expression levels did not meet the gene filtering criteria. [file bcr1637-S1.pdf]

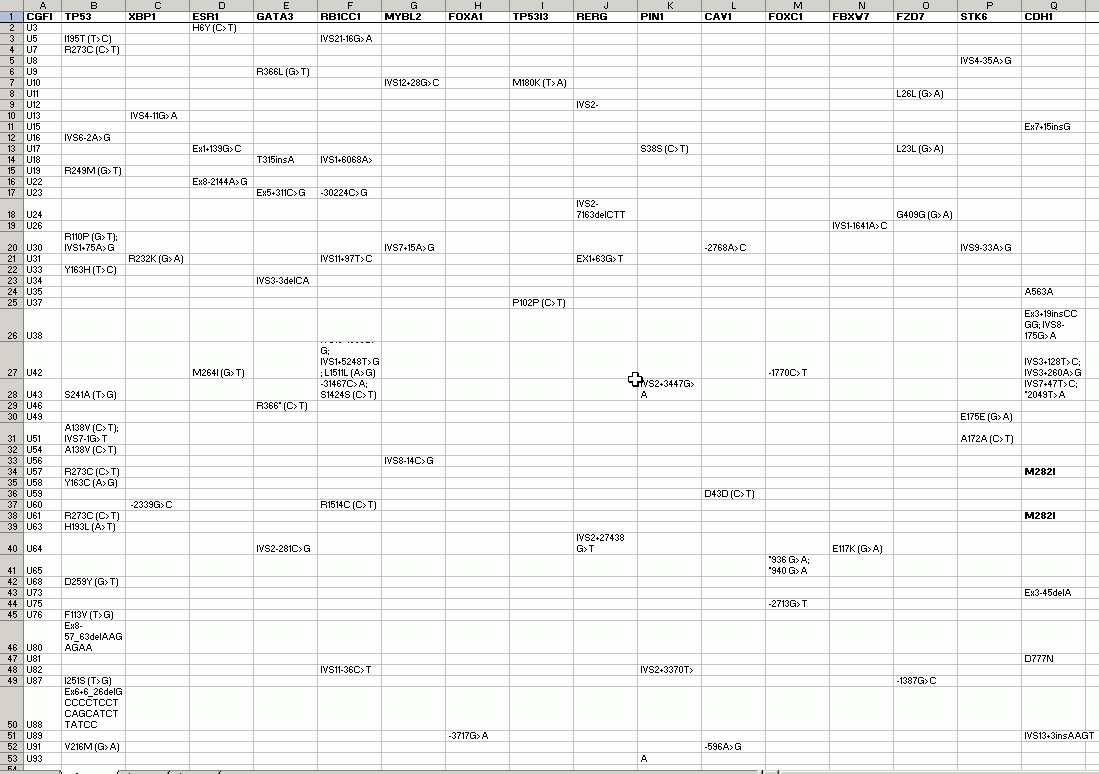

Supplement: Additional file 2 — A doc file in which observed somatic alterations are reported by individual breast cancer tissue sample (n = 53). Somatic alterations previously reported in TP53 and GATA3 are highlighted [4-6,29] (Langerod and coworkers, unpublished data). [file bcr1637-S2.doc]
